# Amyloid‐ß oligomer Aß*56 is associated with Alzheimer’s dementia independently of amyloid pathology

**DOI:** 10.1002/alz.095043

**Published:** 2025-01-09

**Authors:** Peng Liu, Ian Lapcinski, Karen Ashe

**Affiliations:** ^1^ University of Minnesota, Minneapolis, MN USA

## Abstract

**Background:**

A current hypothesis of the etiology of Alzheimer’s disease (AD) suggests that soluble amyloid‐ß (Aß) oligomers (Aßo) play a more significant role than amyloid plaques. Among the numerous Aßo discovered in the brain, Aß*56 has been shown to be associated with aging and cognitive dysfunction in mice, dogs, and humans, and impair memory in rodents. Evidence from our recent study indicates that Aß*56 produced from Tg2576 mice modeling AD is a ∼56‐kDa, SDS‐stable, A11 (anti‐amyloid oligomer antibody)‐reactive, water‐soluble oligomer that impairs memory in healthy wild‐type mice; and that there exist at least two Aß*56 variants—Aß(40)*56 and Aß(42)*56—that contain canonical Aß(1‐40) and Aß(1‐42), respectively, in AD mouse models. In addition, Aß*56 appears prior to the formation of amyloid plaques in Tg2576 mice, suggesting that the biogenesis of Aß*56 is independent on plaques. Here, we extend the characterization of this non‐plaque‐dependent oligomer to human AD cases and investigate the relationship between Aß*56 and cognitive dysfunction in AD dementia.

**Method:**

We analyzed AD‐affected, inferior temporal gyrus (ITG) postmortem specimens from de‐identified elderly individuals enrolled in the Religious Orders Study/Memory and Aging Project. This cohort consisted of cognitively normal (CN) individuals, individuals with mild cognitive impairment (MCI), and individuals with AD dementia (*N* = 19‐23 per group) that were age‐matched, sex‐balanced, and contained comparable amyloid plaque loads in the ITG. Using immunoprecipitation/western blotting coupled with densitometry‐based semi‐quantitative analysis, we measured levels of Aß(40)*56 and Aß(42)*56. We then compared levels of Aß*56 variants between different clinical diagnoses and correlated them to cognitive assessments.

**Result:**

We showed that both levels of Aß(40)*56 and Aß(42)*56 are elevated in individuals with AD dementia compared to CN individuals and individuals with MCI. In addition, we showed that levels of Aß*56 variants are correlated to mini‐mental state examination scores and severity of impairment in global cognitive function, episodic memory, and semantic memory, but not working memory.

**Conclusion:**

Our data suggest an association between Aß*56 to AD dementia in elderly human brains that is independent of amyloid pathology. Such findings support the use of Aß*56 as a molecular marker for AD diagnosis.

